# Results of the Pinhole Test Correlate with Hybrid Contact Lens Visual Acuity in Patients with Visual Impairment due to Corneal Diseases

**DOI:** 10.1155/2022/4932856

**Published:** 2022-06-24

**Authors:** Piotr Kanclerz

**Affiliations:** ^1^Department of Ophthalmology, Hygeia Clinic, Gdansk, Poland; ^2^Helsinki Retina Research Group, University of Helsinki, Helsinki, Finland

## Abstract

**Introduction:**

Trial rigid lens fitting is considered the best approach to determine whether the correction of residual defocus and irregular astigmatism might improve the visual acuity in patients with corneal disorders including keratoconus. This study aimed to analyze the correlation between hybrid lenses and pinhole visual acuity (VA).

**Methods:**

Patients undergoing hybrid contact lens fitting at the Hygeia Clinic, Poland, were included. The VA of each patient was assessed as decimal Snellen fractions under the following conditions: (i) uncorrected VA, (ii) VA with spherocylindrical correction (i.e., corrected distance VA), (iii) VA with a single 1.2 mm pinhole occluder, and (iv) VA with the best-fitted hybrid contact lens. Pearson's correlation coefficient *r* was used to assess correlations among variables.

**Results:**

This study involved 29 eyes of 19 patients, mainly with advanced keratoconus. The uncorrected VA was 0.11 ± 0.10. The pinhole test provided significantly improved VA over corrected distance VA (0.51 ± 0.29 vs. 0.31 ± 0.20, respectively; *p* < 0.0001). Similarly, the fitted hybrid contact lenses provided improved VA over corrected distance VA (0.66 ± 0.26 vs. 0.31 ± 0.20, respectively; *p* < 0.0001). The pinhole VA was strongly correlated with the hybrid contact lens VA (*r* = 0.8135; 95% CI: 0.61–0.92; *p* < 0.0001). The improvement in the pinhole test over corrected distance VA was moderately correlated with the improvement with the fitted lens over corrected distance VA (*r* = 0.6269; 95% confidence interval (CI): 0.32–0.80; *p*=0.0005).

**Conclusions:**

A significant improvement in VA with the pinhole test is a simple predictor of general improvement with hybrid contact lenses. The pinhole test should be used in patients with corneal diseases such as keratoconus to determine whether optical aberrations associated with the disease cause their visual impairment.

## 1. Introduction

Ectatic corneal diseases are a group of conditions characterized by progressive corneal thinning and bulging; several phenotypes of which keratoconus is the most common [1]. Keratoconus can present with various topographic patterns, including round, oval, superior steep, inferior steep, irregular, inferior-steep asymmetric bow tie, superior-steep asymmetric bow tie, and symmetric or asymmetric bow tie. The development of interventions such as cornea crosslinking, photorefractive keratectomy, intrastromal corneal ring segment implantations, and combined treatment provide clinicians with a range of treatment options for visual rehabilitation in patients with keratoconus [2–4]. Notably, combining different protocols of crosslinking and refractive treatment (CXL+), performed either simultaneously or sequentially, has been found to improve the visual function and halt the progression of keratoconus [5]. Still, in patients with keratoconus, it is commonly difficult to achieve satisfactory vision with spectacles due to various factors including high irregular astigmatism and significant anisometropia [6].

The main mode of visual rehabilitation for keratoconus are rigid gas permeable (RGP) contact lenses, which are mostly worn successfully with good visual acuity [7]. In the Collaborative Longitudinal Evaluation of Keratoconus Study, sixty-five percent of the patients wore RGP contact lenses and most of them (73%) reported that their lenses are comfortable [8]. Long-term studies have shown that most patients are fitted with contact lenses when the vision can no longer be corrected to at least 20/30 in glasses [9]. RGP lenses have the benefit of masking corneal irregularities, thus providing a regular anterior refractive surface [10]. Therefore, they have been also successfully used to treat other corneal ectasias, including irregular corneas following photorefractive surgery, penetrating keratoplasty, and corneal dystrophies [10–12]. Comparative studies demonstrated that specialty design contact lenses, new design scleral lenses, and hybrid lenses have better patients' comfort [8] levels than that by conventional RGP lenses [13].

The pinhole occluder, which is an opaque disc with at least one small hole can be used to evaluate whether reduced vision is caused by a refractive error [14]. If this is the case, the pinhole will improve the visual acuity (VA). Worse vision might indicate macular diseases or lens opacities, while no change might indicate amblyopia. The pinhole test is used mainly in adults and older children [15, 16]; it is commonly performed in under-resourced settings [14, 17]; however, the use of this test has been criticized for inaccurate estimation of postrefractive VA [18, 19]. Still, the World Health Organization recommends the use of the pinhole test in the rapid assessment of avoidable blindness survey to distinguish refractive errors and conditions that are not correctable with eyeglasses in the adult [20].

Trial rigid lens fitting is considered the best approach to determine whether the correction of residual defocus and irregular astigmatism might improve VA in keratoconus patients [21]. Still, trial rigid lens fitting is not performed in every practice and might not be readily available in some areas. This study aimed to analyze the correlation between hybrid lens and pinhole VA.

## 2. Methods

This study enrolled patients at Hygeia Clinic, Gdansk, Poland, between November 2015 and March 2021. Patients with corneal diseases decreasing corrected distance VA and admitted for hybrid contact lens fitting were included. The Hygeia Clinic routinely uses UltraHealth (Synergeyes; Carlsbad, CA) hybrid contact lenses, as they provide better vision and contrast sensitivity [22, 23] and higher vision-related quality of life and patient satisfaction [24] compared to RGP lenses. The hybrid lens design allows for better lens centration than in RGP lenses; lens centration is known to be an important factor for the correction of the high-order aberrations [25]. Moreover, their smaller size makes them easier to apply and remove than scleral contact lenses [10]. The UltraHealth contact lens has a diameter of 14.5 mm, and consists of a rigid gas permeable center (petrafocon A; oxygen permeability 130 Dk) and silicone-hydrogel skirt (hem-larafilcon A; oxygen permeability, measured as a function of diffusivity (*D*), solubility (*k*), and lens thickness (*t)*: 84 *Dk*/*t*). The central 6.5 mm reverse geometry optic zone moves to a steeper reverse geometry lift curve that enables the rigid center to vault the central cornea [26]. The lens is available in powers −20.0 to +10.0 dioptre (*D*), with a vault of 50 to 550 *μ*m in 50 *μ*m steps and four skirt curves.

**Figure 1:**
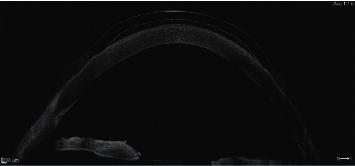
Optical coherence tomography with Revo (Optopol Technologies; Zawiercie, Poland) was performed during hybrid contact lens fitting. Fitting was performed to ensure the central vault of the rigid part provided adequate clearance over the cornea.

Before hybrid lens fitting, a slit lamp examination, corneal topography, and anterior segment optical coherence tomography were performed [27]. Keratoconus was classified according to the Red Temática de Investigación Cooperativa en Salud (RETICS) classification [28]. The VA of each patient was recorded in decimal Snellen fractions and assessed under the following conditions: (i) uncorrected VA, (ii) VA with spherocylindrical correction (i.e., corrected distance VA), (iii) VA with a single 1.2 mm pinhole occluder, and (iv) VA with the best-fitted hybrid contact lens. The 1.2 mm aperture pinhole occluder was selected as it is widely used in trial framesets and does not decrease VA in high refractive errors compared to smaller pinhole occluders. Optimal fitting was performed using optical coherence tomography to ensure the central vault of the rigid part provided adequate clearance over the cornea under the slit lamp (Figure 1) [29].

Statistical analyses were conducted using the Medcalc Statistical Software v.14.0 (Ostend, Belgium). The results are presented as the mean ± standard deviation. Parametric test assumptions were checked with the Kolmogorov–Smirnov test. The *t*-test and the Mann–Whitney *U* test were used to compare groups. Pearson's correlation coefficient (*r*) was used to assess the linear correlation between variables; values between 0 and 0.3 were considered as weak positive, between 0.3 and 0.7 as moderate positive, while between 0.7 and 1.0 as strong positive linear relationships [30]. Correlations and intergroup differences with *p* values of less than 0.05 were considered statistically significant.

## 3. Results

Twenty-nine eyes from 19 individuals (26.7% female) were assessed with an average age of 34.1 ± 7.2 years. Indications for contact lens fitting included keratoconus (23 eyes; 16 eyes RETICS Grade IV and 7 eyes RETICS Grade IV+), map-dot-fingerprint corneal dystrophy (2 eyes), pellucid marginal degeneration (1 eye), corneal scar and irregularity following bacterial keratitis in childhood (1 eye), and irregular cornea following penetrating keratoplasty (2 eyes). All patients had a crystalline lens and manifested no retinal pathologies. The manifest refractive sphere was −2.41 ± 2.95 D, with a mean refractive cylinder of −3.27 ± 1.94 D. The mean maximum keratometry was 54.10 ± 11.66 D, and the minimum radius of the best fit sphere was 6.23 ± 1.00 mm. The mean central corneal thickness was 473 ± 113 *μ*m.

**Figure 2:**
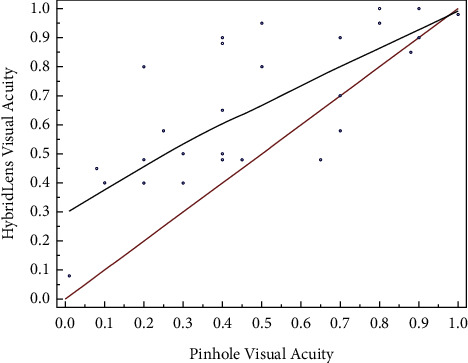
Correlation between measurements of hybrid contact lens visual acuity and pinhole acuity.

The uncorrected VA was 0.11 ± 0.10, and the mean corrected distance VA was 0.31 ± 0.20. The mean vault of the fitted contact lenses was 256 ± 143 *μ*m, providing the recommended 50–100 *μ*m lens clearance. The mean optical power of the fitted lenses was −5.36 ± 3.43 D. Seventeen fitted lenses had a flat curve, eleven had a medium curve, and one had a steep-skirt curve. The pinhole test provided significantly improved VA over corrected distance VA (0.51 ± 0.29 vs. 0.31 ± 0.20, respectively; *p* < 0.0001). Similarly, the fitted hybrid contact lenses provided improved VA over corrected distance VA (0.66 ± 0.26 vs. 0.31 ± 0.20, respectively; *p* < 0.0001). The pinhole VA was strongly correlated with the hybrid contact lens VA (*r* *=* 0.8135; 95% CI: 0.61–0.92; *p* < 0.0001; Figure 2). The improvement in the pinhole test over corrected distance VA was moderately correlated with the improvement with the fitted lens over corrected distance VA (*r* = 0.6269; 95% confidence interval (CI): 0.32–0.80; *p*=0.0005).

## 4. Discussion

Pinhole occluders can improve VA by reducing retinal blur presented by narrowing the beam of light entering the eye [31, 32], cutting off peripheral aberration [33], and increasing the depth of focus [34, 35]. Therefore, contact lenses, corneal inlays, and intraocular lenses employing the pinhole principle have been designed and are commercially available [13, 36, 37]. However, pinhole occluders can reduce the quantity of light reaching the retina [32], causing diffraction blurring and narrowing of the visual field [38]. The pinhole test is often used in clinical practice, however, the utility of the pinhole test in clinical studies has not been extensively described [39, 40]. Kumar et al. found the magnitude of improvement in the pinhole test is correlated with the magnitude of spherical equivalent refraction (Spearman's *ρ* = 0.68, *p* < 0.0001) and concluded that the pinhole occlusion is a valid gauge of refractive error in rapid assessment of avoidable blindness surveys [17]. In addition, Melki et al. suggested that the pinhole test is a simple and reliable method for estimating visual outcomes after uncomplicated cataract surgery [41]. Furthermore, Lowenstein et al. found that the pinhole test reduced the rate of patients with false-positive results in uncorrected VA screening, who would have unnecessarily undergone a complete ophthalmological examination [39] In this study, the pinhole VA was slightly worse than hybrid lens VA. This is concordant to what was reported by Eagan et al. who noted that the pinhole test underestimated the postrefractive VA by 6 letters on the Early Treatment Diabetic Retinopathy Study chart [18].

The main limitation of the study is its small sample size, reflecting the very low prevalence of keratoconus in Poland [42]. Hashemi et al. performed a meta-analysis of the currently published studies and found an overall prevalence of 138 per 100,000 population (95% confidence interval (CI): 114–162 per 100,000) based on results of 7,158,241 participants [43]. However, the real prevalence of keratoconus is unclear because of geographical, genetic, environmental, and cultural factors and variability in the diagnostic criteria and procedures used [42]. The prevalence of keratoconus in Central and Northern Europe is presumably lower than the aforementioned global overall prevalence. For example, a Russian study reported the prevalence of keratoconus as 0.2–0.4 per 100,000 [44], while a Macedonian study reported it as 6.8 per 100,000 [45]. Medical coding database studies have found its prevalence to be 30 per 100,000 in Finland [46] and 44–86 per 100,000 in Denmark [47, 48]. However, the number of patients undergoing rigid contact lens fitting is significantly smaller than the prevalence, as those at an early stage of the disease might be satisfied with spherocylindrical VA. Although our clinic is potentially the largest in the region that performs hybrid contact lens fitting, a larger study is required to confirm our findings.

In conclusion, a significant improvement in VA with the pinhole test can be considered a general predictor of improvement with hybrid contact lenses. While access to RGP or hybrid contact lens fitting might be limited in some regions, the pinhole test is fast and inexpensive and is suitable for routine use in patients with corneal disorders. The use of the pinhole test could be considered in patients with corneal diseases, i.e., keratoconus to investigate whether the optical aberrations associated with the disease are the reason for visual impairment.

## Data Availability

The data used to support the findings of this study are available from the corresponding author on request.
